# Anti-leukemic activity of axitinib against cells harboring the BCR-ABL T315I point mutation

**DOI:** 10.1186/s13045-015-0190-9

**Published:** 2015-08-04

**Authors:** Seiichi Okabe, Tetsuzo Tauchi, Yuko Tanaka, Juri Sakuta, Kazuma Ohyashiki

**Affiliations:** Department of Hematology, Tokyo Medical University, 6-7-1 Nishi-shinjuku, Shinjuku-ku, Tokyo 160-0023 Japan

**Keywords:** Axitinib, Resistant cell, Ponatinib, T315I, Compound mutation

## Abstract

The BCR-ABL; breakpoint cluster region-Abelson point mutation T315I is resistant to ABL tyrosine kinase inhibitors. However, axitinib, a vascular endothelial growth factor receptor inhibitor, is effective against this mutation. In this study, we investigated axitinib activity against ponatinib-resistant cells and found that axitinib inhibited cellular growth and apoptosis in Ba/F3 T315I-mutant cells and T315I-mutant primary samples, but not in ponatinib-resistant Ba/F3 cells and primary samples. Thus, an alternative strategy may be required to improve the prognosis of Philadelphia-chromosome-positive leukemia patients harboring BCR-ABL point mutations.

## Letters to the editor

The BCR-ABL1 fusion gene is a causative oncogene in chronic myeloid leukemia (CML) and 30–50 % of acute lymphoblastic leukemia cases [1, 2]. Although ABL tyrosine kinase inhibitors (ABL TKIs) such as imatinib, nilotinib, dasatinib, and bosutinib have improved CML treatment [3], such therapies cannot cure patients with Philadelphia chromosome (Ph)-positive leukemia because of leukemia stem cells [4]. Moreover, some patients develop BCR-ABL point mutations and become resistant to ABL TKI therapy [5]. In particular, the ABL kinase domain mutation T315I is resistant to imatinib and second-generation ABL TKIs (e.g., nilotinib, dasatinib, and bosutinib). Accordingly, this mutation is often found in patients with TKI-resistant disease [6]. A third-generation ABL TKI, ponatinib, and omacetaxine which is a semisynthetic form of homoharringtonine, was recently developed [7]. Ponatinib is a potent oral tyrosine kinase inhibitor that affects both unmutated and mutated BCR-ABL [8]; it is effective against T315I-mutant cells and has been approved for TKI-resistant or intolerant CML and Ph-positive ALL patients. Omacetaxine is approved for the treatment of chronic or accelerated-phase CML refractory to TKIs [7].

**Fig. 1:**
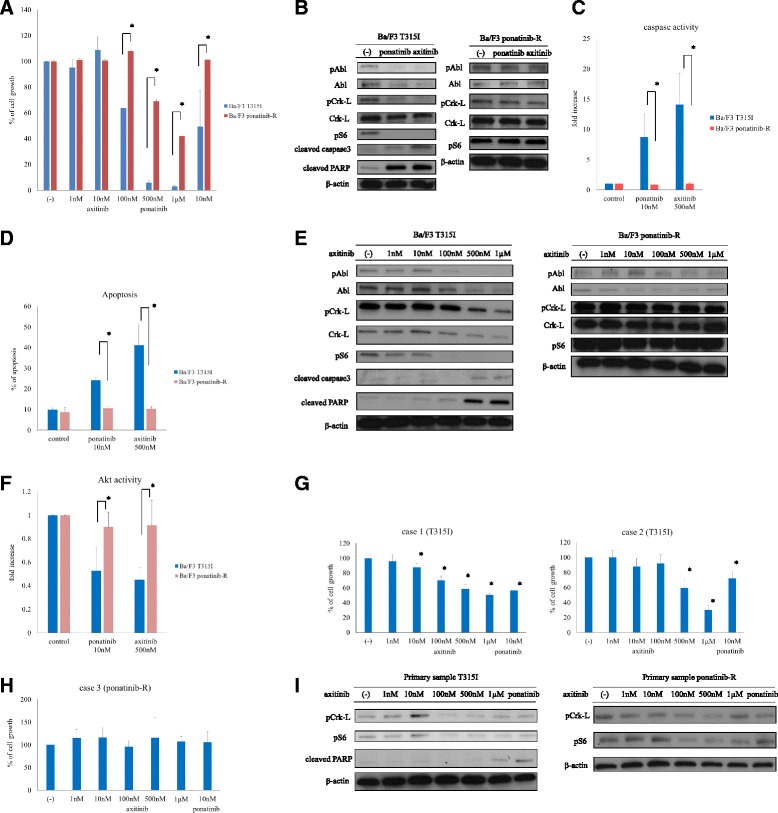
Growth inhibition and cellular signaling following axitinib/ponatinib treatment in T315I-mutant and compound-mutant cells. **a** Ba/F3 T315I or Ba/F3 ponatinib-resistant (Ba/F3 ponatinib-R) cells were exposed to axitinib or ponatinib for 72 h at the indicated concentrations and subjected to quantitative cell proliferation analysis. Each result is presented as the mean percentage of proliferation relative to unexposed control cultures. *P < 0.05 compared to T315I cells. **b** Ba/F3 T315I or Ba/F3 ponatinib-R cells were treated with ponatinib (10 nM) or axitinib (500 nM) for 24 h. Total cell extracts were examined via immunoblot analysis with anti-phospho ABL, phospho-Crk-L, phospho-S6, cleaved caspase 3, cleaved PARP, ABL, Crk-L, and β-actin antibodies. **c** Caspase activity was analyzed using an ApoAlert® Caspase-3 Colorimetric Assay Kit (Takara Bio Inc. Otsu, Shiga, Japan) according to the manufacturer’s protocol. Data represent three independent sets of experiments. *P < 0.05 compared to Ba/F3 T315I and Ba/F3 ponatinib-R cells. **d** Apoptosis in Ph-positive cell lines was assayed using a FITC Annexin V Apoptosis Detection Kit I™ (BD Pharmingen, San Jose, CA, USA). The experiments were performed in triplicate. **e** Ba/F3 T315I or Ba/F3 ponatinib-R cells were treated with axitinib at the indicated concentrations for 24 h. Total cell extracts were examined via immunoblot analysis with anti-phospho ABL, phospho-Crk-L, phospho-S6, cleaved caspase 3, cleaved PARP, ABL, Crk-L, and β-actin antibodies. **f** Akt activity was analyzed using a phospho-AKT 1/2/3 (Ser473) InstantOne™ enzyme-linked immunosorbent assay kit (Affymetrix, Cleveland, OH, USA) according to the manufacturer’s protocol. Data represent three independent sets of experiments. *P < 0.05 compared to Ba/F3 T315I and Ba/F3 ponatinib-R cells. **g**, **h** T315I-positive or compound-mutant primary cells were subjected to quantitative cell proliferation analysis after a 72-h exposure to axitinib or ponatinib. Each result is presented as the mean percentage of proliferation relative to unexposed control cultures. *P < 0.05 compared to control. **i** T315I-positive or compound-mutant primary cells were treated with axitinib at the indicated concentrations for 24 h. Total cell extracts were examined via immunoblot analysis with anti-phospho-Crk-L, phospho-S6, cleaved PARP, and β-actin antibodies

Recently, the vascular endothelial growth factor receptor (VEGFR) inhibitor axitinib was found to exhibit anti-leukemic activity against T315I-mutant disease. In the comparative effectiveness of axitinib versus sorafenib in advanced renal cell carcinoma (AXIS) trial [9], axitinib improved progression-free survival (PFS) compared to sorafenib, which is an all-multikinase inhibitor that blocks angiogenesis targets [10], in patients with advanced renal cell carcinoma (RCC). Axitinib was approved for the treatment of advanced RCC. Axitinib is an orally active and potent TKI of VEGFRs 1, 2, and 3 and inhibits BCR-ABL1, especially the T315I variant, via a distinct binding conformation [11]. In this study, we investigated whether axitinib could suppress ponatinib-resistant compound-mutant cells harboring the T315I mutation. A 72-h axitinib treatment inhibited the growth of Ba/F3 T315I cells (Fig. 1a). Immunoblot analysis of axitinib-treated cells revealed dose-dependent decreases in BCR-ABL, the downstream molecule Crk-L, and ribosomal S6 protein phosphorylation and increases in caspase 3 and Poly (ADP-ribose) polymerase (PARP) activity (Fig. 1b, c, e). Ponatinib and axitinib also induced apoptosis, significantly increased caspase activity (Fig. 1d), and reduced Akt activity (Fig. 1f).

In contrast, clinically available concentrations of axitinib did not inhibit the growth of ponatinib-resistant Ba/F3 cells (Fig. 1a). Immunoblot analysis revealed that BCR-ABL, Crk-L, and S6 kinase phosphorylation were not inhibited by axitinib or ponatinib (Fig. 1b, e). Similarly, no increase in caspase activity or decrease in Akt activity was observed following axitinib treatment (Fig. 1c, f), and neither ponatinib nor axitinib affected apoptosis in these cells (Fig. 1d).

We next evaluated primary T315I-mutant and ponatinib-resistant compound-mutant samples. Axitinib potently inhibited the growth of T315I-mutant primary cells in a dose-dependent manner (Fig. 1g). Immunoblot analysis further revealed reduced Crk-L and S6 kinase phosphorylation after axitinib or ponatinib treatment (Fig. 1i). In contrast, the growth of ponatinib-resistant primary cells was not affected by ponatinib or axitinib (Fig. 1h). Immunoblotting revealed that neither ponatinib nor axitinib affected the phosphorylation of Crk-L and S6 kinase in ponatinib-resistant cells (Fig. 1i).

In CML, ABL TKI resistance is frequently caused by ABL kinase domain mutations. The T315I mutation is resistant to all ABL TKIs except ponatinib. However, we previously described ponatinib-resistant cells resulting from a BCR-ABL compound mutation [12]. Although axitinib, which is currently being investigated for efficacy in patients with Ph-positive T315I-mutant leukemia, induced apoptosis in T315I-mutant cells, it was ineffective against cells with a compound mutation including T315I. An alternative strategy will be required to improve the prognosis of patients with Ph-positive, BCR-ABL-mutant leukemia. Current evidence to direct the management of ABL TKI-resistant disease, particularly those harboring T315I and compound mutations, is limited. New molecular-targeted drugs and an understanding of ABL TKI resistance mechanisms are required to apply an appropriate therapeutic approach.

## Abbreviations

CML: chronic myeloid leukemia
Ph: Philadelphia chromosome
TKI: tyrosine kinase inhibitor
VEGFR: vascular endothelial growth factor receptor
